# Real-time precision detection algorithm for jellyfish stings in neural computing, featuring adaptive deep learning enhanced by an advanced YOLOv4 framework

**DOI:** 10.3389/fnbot.2024.1375886

**Published:** 2024-05-23

**Authors:** Chao Zhu, Hua Feng, Liang Xu

**Affiliations:** Emergency Department of Qinhuangdao First Hospital, Qinhuangdao, Hebei, China

**Keywords:** YOLOv4, attention mechanism, PID, jellyfish stings, intelligent robotics, computer vision

## Abstract

**Introduction:**

Sea jellyfish stings pose a threat to human health, and traditional detection methods face challenges in terms of accuracy and real-time capabilities.

**Methods:**

To address this, we propose a novel algorithm that integrates YOLOv4 object detection, an attention mechanism, and PID control. We enhance YOLOv4 to improve the accuracy and real-time performance of detection. Additionally, we introduce an attention mechanism to automatically focus on critical areas of sea jellyfish stings, enhancing detection precision. Ultimately, utilizing the PID control algorithm, we achieve adaptive adjustments in the robot's movements and posture based on the detection results. Extensive experimental evaluations using a real sea jellyfish sting image dataset demonstrate significant improvements in accuracy and real-time performance using our proposed algorithm. Compared to traditional methods, our algorithm more accurately detects sea jellyfish stings and dynamically adjusts the robot's actions in real-time, maximizing protection for human health.

**Results and discussion:**

The significance of this research lies in providing an efficient and accurate sea jellyfish sting detection algorithm for intelligent robot systems. The algorithm exhibits notable improvements in real-time capabilities and precision, aiding robot systems in better identifying and addressing sea jellyfish stings, thereby safeguarding human health. Moreover, the algorithm possesses a certain level of generality and can be applied to other applications in target detection and adaptive control, offering broad prospects for diverse applications.

## 1 Introduction

The development of intelligent robots has been widely applied across various fields, including target detection and adaptive control (Martin-Abadal et al., 2020). In tasks such as marine exploration and rescue missions, detecting sea Jellyfish stings is crucial due to the threat they pose to human health. However, traditional detection methods face challenges in terms of accuracy and real-time capabilities, necessitating the development of a new algorithm (Cunha and Dinis-Oliveira, 2022). The purpose of this paper is to propose an adaptive intelligent robot algorithm for real-time and accurate sea Jellyfish sting detection, based on an improved Yolov4, attention mechanism, and PID control. This algorithm aims to enhance the accuracy and real-time performance of sea Jellyfish sting detection, thereby better safeguarding human health (Cunha and Dinis-Oliveira, 2022). Here are five commonly used deep learning or machine learning models in the fields of target detection and adaptive control:

YOLO (You Only Look Once) (Gao M. et al., 2021) is a fast and real-time object detection model. It employs a single neural network to perform object detection in a single forward pass, making it suitable for applications with high real-time requirements. YOLO's network structure is relatively simple, and both training and inference processes are efficient. By dividing the image into a grid, with each grid predicting the bounding box and category of the target, YOLO can capture global contextual information. However, YOLO exhibits lower detection accuracy for small and dense targets, and its localization precision is limited.

Faster R-CNN (Region-based Convolutional Neural Network) (Zeng et al., 2021) is an object detection model with high detection accuracy. It achieves object detection through two main steps: extracting candidate regions and classifying and locating these regions. Faster R-CNN excels in detection accuracy and can handle various target sizes and densities. However, due to the need for multiple steps and complex computations, Faster R-CNN has a relatively slower speed and is not suitable for real-time applications.

SSD (Single Shot MultiBox Detector) (Ma et al., 2021) is a fast object detection model suitable for real-time applications. SSD detects targets by applying a convolutional sliding window on feature maps of different scales. It has good detection speed and high accuracy, adapting well to targets of different sizes. However, compared to other models, SSD's detection accuracy for small targets is relatively lower.

RetinaNet (Liu et al., 2023) is an object detection model that performs well in handling small targets. It introduces a novel loss function that balances samples with different target sizes. It exhibits good performance in detecting small targets, effectively addressing the issue of small targets being easily overlooked. However, its detection accuracy is relatively lower when dealing with dense and large targets.

Mask R-CNN (Nie et al., 2020) is an object detection model capable of pixel-level segmentation of targets. In addition to detecting the bounding box and category of targets, Mask R-CNN can generate precise masks for targets. This makes Mask R-CNN highly useful when detailed target segmentation information is required. However, due to the need for pixel-level predictions, Mask R-CNN has a relatively slower speed.

The following are three related research directions:

Improving small object detection accuracy in real-time object detection models. Real-time object detection plays a crucial role in various application domains, but current real-time models face challenges in achieving high accuracy for small object detection (Mahaur et al., 2023). To enhance the small object detection accuracy in real-time object detection models, research can focus on the following aspects: Firstly, improving feature representation capabilities. Secondly, designing more refined object detection loss functions. Existing object detection loss functions may have issues with small objects as they tend to prioritize larger targets (Khamassi et al., 2023). By researching and improving in the above directions, the performance of real-time object detection models in small object detection accuracy can be enhanced, expanding their applicability to a wider range of real-world scenarios (Zhang et al., 2022).

Integrating multimodal information in object detection models (Chen et al., 2019). Object detection is typically based on image data, but in some application scenarios, combining multimodal information from other sensors may provide more accurate and comprehensive object detection results (Gao W. et al., 2021). Therefore, researching object detection models that integrate multimodal information is a promising direction. One approach is to fuse image data with other sensor data to improve detection accuracy and robustness (Wu et al., 2021).

Designing and optimizing lightweight object detection models. In resource-constrained scenarios like embedded devices or mobile platforms, there is a demand for object detection models with small model sizes and low computational complexity while maintaining high detection accuracy (Han et al., 2022). Therefore, designing and optimizing lightweight object detection models is a challenging and practical direction (Li et al., 2018). One approach is to reduce model size and computational complexity through network compression and model pruning (Huang et al., 2018). Exploring the use of lightweight network structures such as MobileNet and ShuffleNet for fine-tuning on object detection tasks is one option. Additionally, techniques like parameter sharing, channel pruning, and quantization can reduce model parameters and computations for designing lightweight object detection models (Lin and Xu, 2023).

Traditional sea Jellyfish sting detection methods face issues in accuracy and real-time capabilities. Therefore, we propose a new algorithm that integrates improved Yolov4, attention mechanism, and PID control to enhance detection accuracy and real-time performance. Firstly, we enhance Yolov4 to improve the accuracy and real-time performance of detection. This involves adjusting network architecture, loss functions, and data augmentation strategies to adapt Yolov4 for sea Jellyfish sting detection tasks. Secondly, we introduce an attention mechanism to automatically focus on critical areas of sea Jellyfish stings, enhancing detection precision. Using attention mechanisms such as SENet or SAM enhances the model's focus on target areas, improving accuracy and robustness. Lastly, we employ the PID control algorithm to achieve adaptive adjustments in the robot's movements and posture based on detection results. The PID control algorithm adjusts parameters in response to error signals, enabling real-time and precise control based on detected sea Jellyfish stings. In the field of sea Jellyfish sting detection, traditional methods face challenges in accuracy and real-time capabilities. Thus, we propose an adaptive intelligent robot algorithm for real-time and accurate sea Jellyfish sting detection, integrating improved Yolov4, attention mechanism, and PID control. This algorithm addresses issues with traditional methods and enhances the ability to protect human health.

- Comprehensive comparison of different object detection models: This paper provides a comprehensive comparison of five commonly used object detection models, namely YOLO, Faster R-CNN, SSD, RetinaNet, and Mask R-CNN. By analyzing their strengths and weaknesses, readers can gain a better understanding of each model's characteristics, enabling them to choose the most suitable model for their specific application scenarios.
- Emphasis on model applicability and limitations: The paper underscores the applicability and limitations of each model. This information assists readers in selecting the most appropriate object detection model based on their individual needs and application contexts. For instance, if real-time performance is a priority, faster models like YOLO or SSD may be preferred. Conversely, if higher detection accuracy is required, Faster R-CNN or RetinaNet might be more suitable.
- Providing a comprehensive understanding of object detection models: The paper offers brief introductions to the principles and features of each model, enabling readers to gain a comprehensive understanding of object detection models. This knowledge empowers readers to delve deeper into the research and application of object detection technology, making informed decisions in practical projects.

## 2 Methodology

### 2.1 Overview of our network

**Figure 1:**
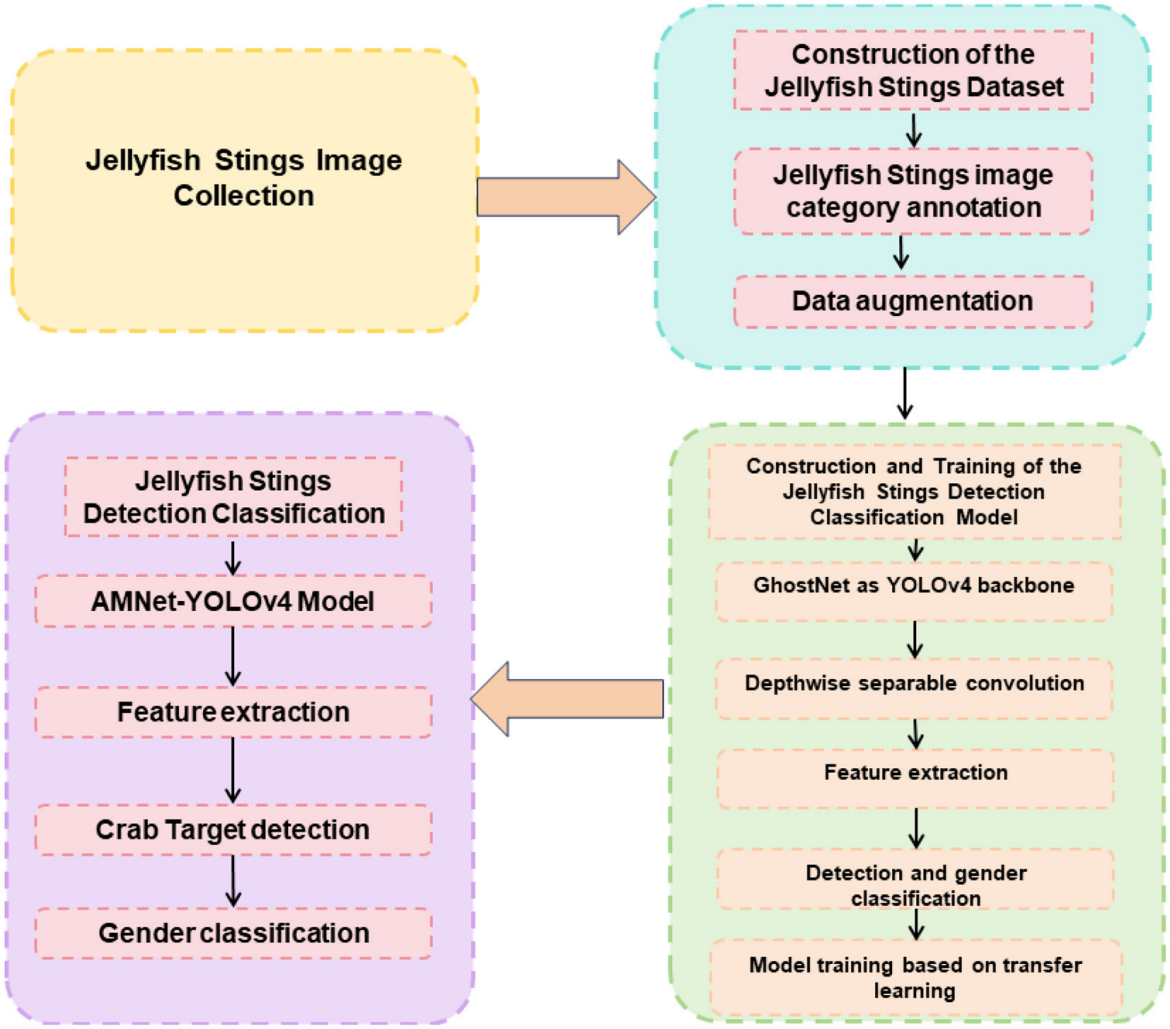
The overall schematic diagram of the proposed model.

The Adaptive Intelligent Robot Real-time Accurate Detection Algorithm for Sea Jellyfish Sting Injuries, based on Improved YOLOv4 and Attention Mechanism combined with PID Control, aims to achieve precise detection and identification of sting injuries in the marine environment. This is accomplished by integrating object detection, attention mechanism, and control algorithms to adaptively adjust the robot's actions in response to changes and errors during the detection process. Figure 1 represents the overall schematic diagram of the proposed model.

Optimize the network structure, training strategies, and loss functions of YOLOv4 to enhance the accuracy and efficiency of the object detection algorithm. Introduce an attention mechanism to enable the algorithm to focus on important image regions, improving detection accuracy and robustness. This can be achieved by adding attention modules to the network or by adjusting feature map weights. Design a PID control algorithm to utilize the error between detection results and expected values to adjust the robot's actions and behaviors. This adaptation is crucial to cope with variations and errors encountered during the detection process.

Overall implementation process:

- Data collection and preparation: Gather images or video data from the marine environment and preprocess it, including tasks such as image enhancement and noise reduction.
- Design of object detection network: Design and enhance the YOLOv4 network structure, involving adjustments to network layers, the introduction of new feature extraction modules, or optimization of loss functions.
- Introduction of attention mechanism: Incorporate an attention mechanism into the object detection network, allowing the model to concentrate on crucial image regions. This can be achieved by adding attention modules or adjusting feature map weights within the network.
- Design of PID control algorithm: Develop a PID control algorithm to dynamically adjust the robot's actions and behaviors based on the error between detection results and expected values. The PID algorithm encompasses proportional, integral, and derivative control parameters.
- Training and optimization: Train the improved network using annotated data and optimize network parameters and attention mechanisms through the iterative process of backpropagation. This optimization is performed iteratively on training and validation sets. Real-time Detection and Feedback:

Deploy the trained model and control algorithm to the intelligent robot for real-time detection and feedback in the marine environment. The robot captures marine images or videos, feeds them into the object detection network for real-time sting injury detection, and adjusts its actions based on the comparison between detection results and expected values. This adaptation allows the robot to accommodate changes and errors encountered during the detection process.

### 2.2 Advanced YOLOv4 model

**Figure 2:**
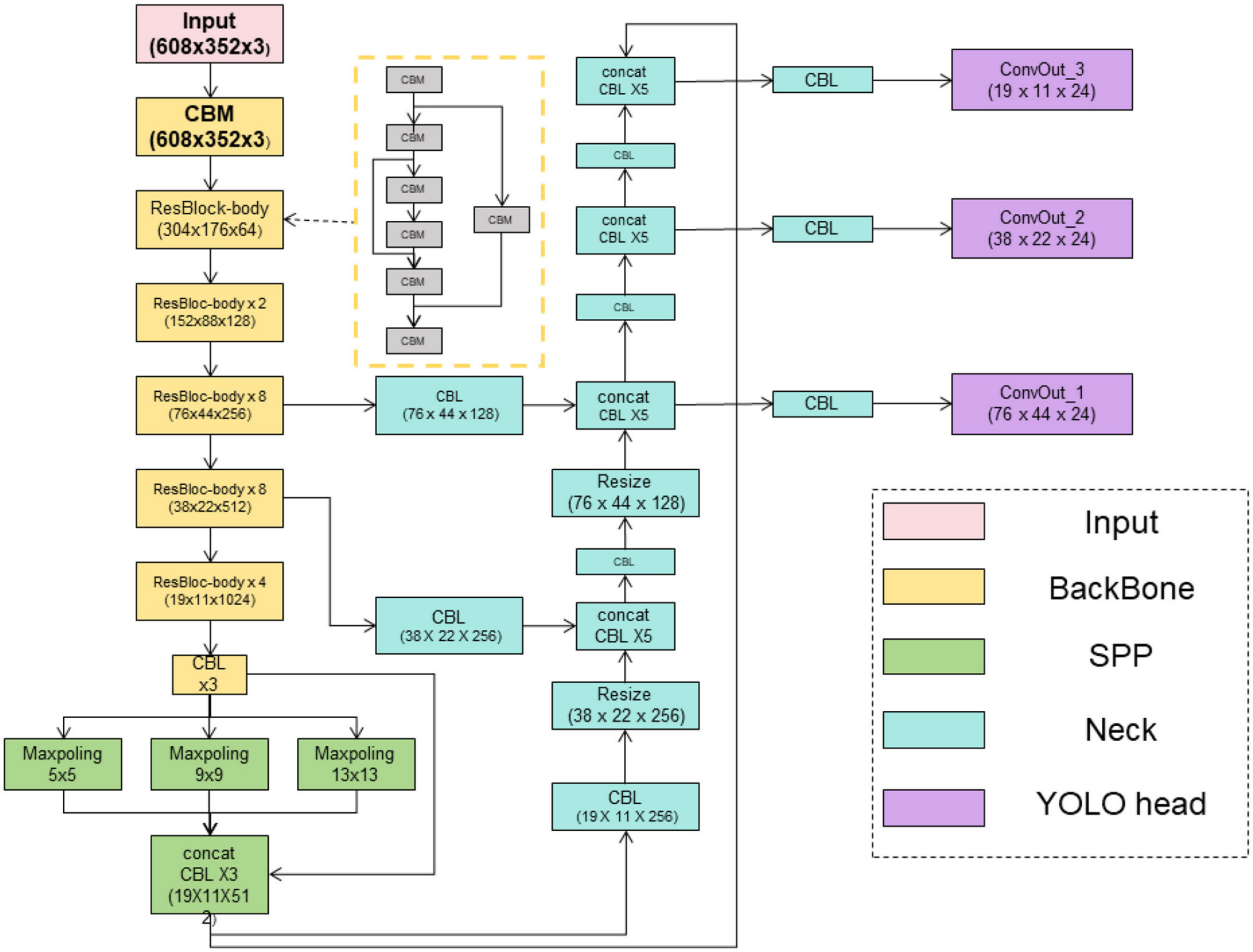
The schematic diagram of the proposed Advanced YOLOv4 model.

Advanced YOLOv4 is an improved version of the traditional YOLOv4 object detection algorithm, designed to enhance detection accuracy and efficiency. The following details the fundamental principles and roles of the Advanced YOLOv4 model in this approach (Roy et al., 2022). Advanced YOLOv4 incorporates a series of improvements, including adjustments to the network structure, optimization of feature extraction modules, enhancement of loss functions, and optimization of training strategies. These improvements aim to enhance the performance and speed of the object detection algorithm (Wang and Liu, 2022). Figure 2 shows the schematic diagram of the proposed Advanced YOLOv4 model.

Network structure adjustments:

Advanced YOLOv4 modifies the YOLOv4 network structure by introducing additional convolutional layers and residual connections, thereby enhancing the network's representational and feature extraction capabilities. Optimization of Feature Extraction Modules:

The model adopts CSPDarknet53 as the primary feature extraction module, combining Cross-Stage Partial connections and the structure of Darknet53. This integration better extracts image features, contributing to improved detection accuracy. Improved Loss Function:

Advanced YOLOv4 utilizes an enhanced loss function known as the Generalized Intersection over Union (GIoU) loss function. This function considers the overlap of target boxes when calculating position and size errors, providing a more accurate measure of target box matching. Optimized Training Strategy:

The model employs a multi-scale training strategy, training the model on images at different scales. This approach enhances the model's adaptability to targets of varying sizes.

“GhostNet” is a lightweight convolutional neural network architecture that introduces “ghost” modules, which use fewer parameters and computational resources in each convolutional layer, thereby achieving higher computational efficiency. In the Advanced YOLOv4 model, we have incorporated “GhostNet” as part of the base network structure to enhance the model's lightweight characteristics, speed up the detection process, and reduce the computational resource requirements of the model.

“Depthwise Separable Convolution” is a type of convolution operation that decomposes standard convolution into two steps: depthwise convolution and pointwise convolution. This decomposition significantly reduces the number of parameters and computational load in the model, thereby improving the model's computational efficiency and speed. In the Advanced YOLOv4 model, we have adopted “Depthwise Separable Convolution” as part of the convolution operations to accelerate the model's inference process and enable faster real-time detection.

Role in the Method: Advanced YOLOv4 plays a crucial role in the Adaptive Intelligent Robot Real-time Accurate Detection Algorithm for Sea Jellyfish Sting Injuries, which combines improved YOLOv4 and attention mechanisms with PID control.

- Improved detection accuracy: Through network structure adjustments and feature extraction module optimization, Advanced YOLOv4 better extracts image features, thereby enhancing the accuracy of object detection. This is crucial for precise detection and identification of sea Jellyfish sting injuries.
  Enhanced detection efficiency: Optimization of the network structure and training strategies in Advanced YOLOv4 contributes to improved speed and efficiency of the object detection algorithm. This is crucial for real-time detection and feedback, enabling intelligent robots to respond promptly to detection results.
- Improved loss function impact: The use of the GIoU loss function in Advanced YOLOv4 contributes to more accurate measurement of target box matching. This aids in improving detection precision and provides more accurate error signals for adaptive control.

The formula for Advanced YOLOv4 is as follows (Equation 1):

Coordinate loss term:


(1) $$\begin{array}{l} \text{coord}\_\text{loss = }\lambda_{\text{coord}}\sum_{i=0}^{S^{2}}\sum_{j=0}^{B}{⊮}_{ij}^{\text{obj}}\left[{\left(x_{i}-{\hat{x}}_{i}\right)}^{\text{2}}\text{+}{\left(y_{i}-{\hat{y}}_{i}\right)}^{\text{2}}\right]\\ \text{ + }\lambda_{\text{coord}}\sum_{i=0}^{S^{2}}\sum_{\text{j=0}}^{B}{⊮}_{ij}^{\text{obj}}\left[{\left(\sqrt{w_{i}}\sqrt{{\hat{w}}_{i}}\right)}^{\text{2}}\text{+}{\left(\sqrt{h_{i}}\sqrt{{\hat{h}}_{i}}\right)}^{\text{2}}\right] \end{array}$$

Among them, λ_coord_ is the weight parameter of the coordinate loss, *S* is the size of the feature map, *B* is the number of bounding boxes predicted for each grid, ${⊮}_{ij}^{\text{obj}}$ represents the indicator function of whether the *j*-th bounding box in the *i*-th grid contains the target, *x*_*i*_, *y*_*i*_ is the *j*-th boundary box in the *i*-th grid The center coordinates of the bounding box, ${\hat{x}}_{i},{ŷ}_{i}$ are the predicted center coordinates of the *j*-th bounding box in the *i*-th grid, *w*_*i*_, *h*_*i*_ are the −*thThewidthandheightofthe*j−*thboundingboxinthei*-th grid, ŵ_*i*_, ĥ_*i*_ are the predicted widths of the *j*-th bounding box in the *i*-th grid and height (Equation 2).

Category loss items:


(2) $$coord_{l}oss\text{=}\sum_{i=0}^{S^{2}}\sum_{j=0}^{B}{⊮}_{ij}^{\text{obj}}{\left(C_{i}-{\hat{C}}_{i}\right)}^{\text{2}}$$

Among them, *C*_*i*_ is the category confidence score of the *j*-th bounding box in the *i*-th grid, and Ĉ_*i*_ is the *j*-th bounding box in the *i*-th grid (Equation 2). Predicted class confidence score.

The final loss function is:


(3) $$\begin{array}{l} \text{L}=\;coord_{l}oss\;+\;conf_{l}oss\;+\;other_{l}oss\; \end{array}$$

This loss function will be optimized during training to minimize the difference between the predicted and ground-truth boxes (Equation 3). By adjusting the weight parameters and optimization algorithm, the performance of the target detection model can be improved.

This formula describes the loss function of Advanced YOLOv4, which includes coordinate loss terms and category loss terms. The coordinate loss term measures the difference between the location and size predictions of the object's bounding box and the ground truth, while the category loss term measures the difference between the class confidence prediction of the object and the ground truth.

In summary, Advanced YOLOv4, through enhancements in network structure, feature extraction modules, loss functions, and training strategies, elevates the performance and speed of the object detection algorithm. It plays a key role in the Adaptive Intelligent Robot Real-time Accurate Detection Algorithm for Sea Jellyfish Sting Injuries, based on improved YOLOv4 and attention mechanisms combined with PID control.

### 2.3 Attention mechanism

Attention Mechanism is a method that simulates human visual or auditory attention and is widely used in deep learning models, especially in Natural Language Processing (NLP) and Computer Vision (CV) tasks (Obeso et al., 2022). The fundamental idea of the attention mechanism is that, given an input sequence and a query (or key information), the model calculates the degree of correlation between each input position and the query. It assigns a weight to each input position, representing the model's focus or importance for different input positions. Then, by taking the weighted sum of the features at input positions using their corresponding weights, the final context representation is obtained (Gao et al., 2020). In NLP tasks, the input sequence can be a sentence or a text sequence, and the query can be a specific word or position. In CV tasks, the input sequence can be the feature map of an image, and the query can be a spatial position or a specific region of the image.

**Figure 3:**
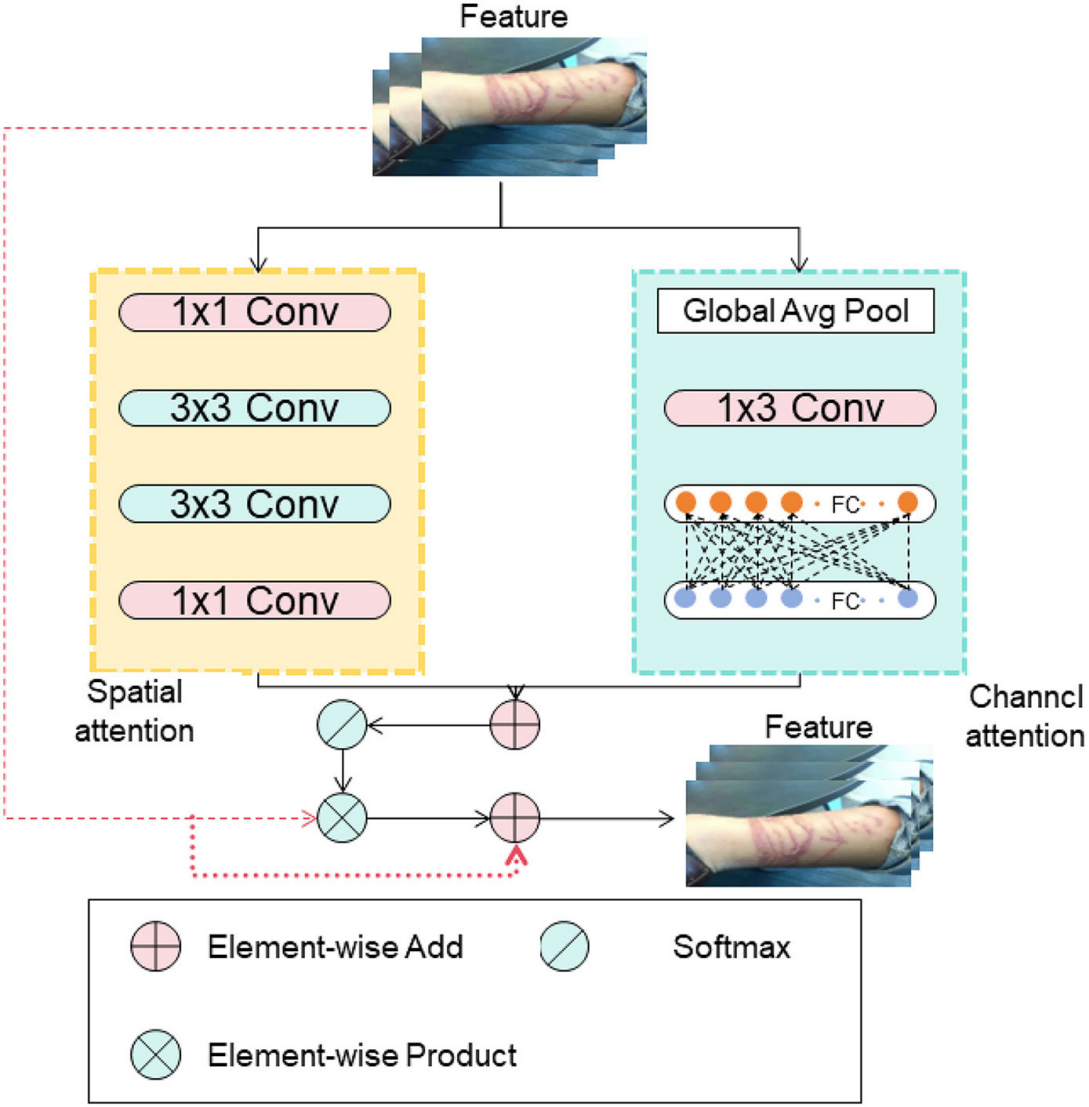
The schematic diagram of the Attention Mechanism.

Figure 3 shows the schematic diagram of the Attention Mechanism.

In attention mechanisms, the most commonly used is soft attention, and its computation process is as follows:

Calculation of correlation between the input sequence and the query: this is done by computing similarity scores between each position in the input sequence and the query, using methods like dot product, scaled dot product, bilinear, or multi-layer perceptron.

Normalization of correlation: to obtain the weight for each position, normalization of the correlation is performed. The softmax function is often used to convert scores into a probability distribution, ensuring that the weights sum up to 1.

Calculation of context representation: the final context representation is obtained by taking the weighted sum of the features in the input sequence using the normalized weights. This context representation can be used for subsequent computations or tasks.

Functions: Attention mechanisms play a crucial role in deep learning models, offering several advantages:

Focus on important information: By calculating the weight for each position, the model can automatically focus on relevant and crucial information in the input sequence related to the query. This enables the model to handle long sequences or large inputs more effectively and extract key features relevant to the task.

Context awareness: Attention mechanisms allow the model to consider information from other positions while processing each position. This context awareness helps improve the model's understanding and generalization capabilities.

Flexibility and interpretability: Attention mechanisms are flexible and can be designed and adjusted according to the requirements of the task. Additionally, the distribution of attention weights provides interpretability, allowing us to understand which parts of the input the model is focusing on.

The formula of the attention mechanism is as follows:


(4) $$\begin{array}{l} \text{Attention}\left(Q,K,V\right)=\text{softmax}\left(\frac{QK^{T}}{\sqrt{d_{k}}}\right)V \end{array}$$

Among them, the explanation of variables is as follows Equation (4):

*Q*: query matrix, indicating the location or information that the model focuses on. *K*: key matrix, representing the position or feature of the input sequence. *V*: value matrix, representing the characteristics of the input sequence. *d*_*k*_: dimension of the key matrix (usually the number of columns of the key matrix). softmax: softmax function, used to convert scores into probability distributions. *T*: Transpose operation, transpose the matrix. The calculation process of the attention mechanism is to do the dot product of the query matrix and the key matrix, then divide it by a scaling factor $\sqrt{d_{k}}$, and finally obtain the weight through the softmax function. These weights are weighted and summed with the value matrix to obtain the final context representation.

Attention mechanisms enable models to dynamically and selectively focus on different parts of a sequence when processing sequential data, thereby enhancing the model's performance and capabilities. It has achieved significant success in various NLP and CV tasks and remains a hot topic in current deep learning research.

### 2.4 PID algorithm

**Figure 4:**
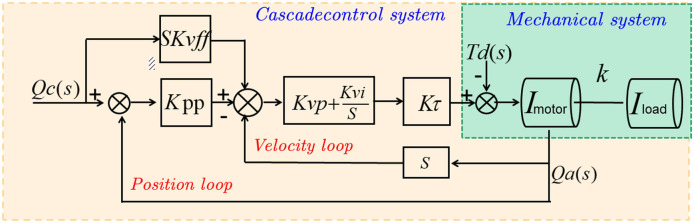
The schematic diagram of the PID algorithm.

The PID algorithm (Proportional-Integral-Derivative) (Vuong and Nguyen, 2023) is a classical control algorithm used for implementing adaptive control systems. The PID algorithm adjusts the controller's output based on the current error, past accumulated error, and rate of change of the error to achieve the desired adjustment of the system's dynamic characteristics (Xu et al., 2023). Figure 4 shows the schematic diagram of the PID algorithm.

The basic principle of the PID algorithm is to continuously adjust the controller's output to minimize the error between the actual system output and the desired output. It consists of three main control components:

Proportional term: The proportional term is directly proportional to the current error and generates a control output proportional to the error magnitude. The proportional term provides a fast response to system changes but may result in steady-state error.

Integral term: The integral term is proportional to the accumulated past errors and is used to handle steady-state errors in the system. The integral term helps eliminate steady-state errors but may lead to overresponse or oscillations.

Derivative term: The derivative term is proportional to the rate of change of the error and is used to predict the future trend of the system. The derivative term helps dampen oscillations and provide a fast response, but it may also result in excessive sensitivity.

The PID algorithm calculates the controller's output by weighted summation of the system's actual error, rate of change of the error, and accumulated error. The formula for the PID algorithm is as follows:


(5) $$u(t)=K_{p}\cdot e(t)+K_{i}\cdot \int_{0}^{t}e(\tau )d\tau +K_{d}\cdot \frac{de(t)}{dt}$$

where Equation (5):

*u*(*t*) is the controller's output at time *t*. *e*(*t*) is the error of the system, defined as the difference between the desired output and the actual output. *K*_*p*_ is the gain coefficient for the proportional term, which adjusts the influence of the proportional control. *K*_*i*_ is the gain coefficient for the integral term, which adjusts the influence of the integral control. *K*_*d*_ is the gain coefficient for the derivative term, which adjusts the influence of the derivative control.

The PID algorithm aims to continuously adjust the controller's output to gradually approach the desired output and maintain it near the setpoint. By properly setting the PID parameters, the system's stability, fast response, and accurate control can be achieved.

## 3 Experiment

### 3.1 Datasets

In this paper, we conduct experiments using four datasets.

COCO dataset (common objects in context): The COCO dataset Sharma (2021) is a widely used large-scale dataset for object detection, segmentation, and captioning tasks. It consists of a diverse collection of images with over 80 common object categories, captured in various contexts. The dataset provides bounding box annotations for object detection, pixel-level segmentations for semantic segmentation, and captions for image captioning. COCO is popular among researchers and used as a benchmark for evaluating object detection and segmentation algorithms.

Pascal VOC dataset (visual object classes): The Pascal VOC dataset Tong and Wu (2023) is another widely used dataset for object detection, segmentation, and classification tasks. It was created for the annual Visual Object Classes challenge and consists of images from 20 different object categories, including animals, vehicles, and common objects. The dataset provides bounding box annotations for object detection, segmentation masks for semantic segmentation, and class labels for classification. Pascal VOC has been widely used for evaluating and comparing various computer vision algorithms.

KITTI dataset: The KITTI dataset Al-refai and Al-refai (2020) is specifically designed for autonomous driving and computer vision tasks related to self-driving cars. It includes various sensor modalities such as stereo cameras, LIDAR, and GPS/IMU data. The dataset contains a large number of annotated images captured from a car-mounted sensor suite, covering scenes from urban environments. It provides annotations for tasks such as object detection, tracking, road segmentation, and depth estimation. The KITTI dataset is commonly used for developing and evaluating algorithms related to autonomous driving and scene understanding.

Open Images dataset: The Open Images dataset Veit et al. (2017) is a large-scale dataset that aims to provide diverse and comprehensive visual data for various computer vision tasks. It contains millions of images from a wide range of categories, covering objects, scenes, and activities. The dataset provides annotations for object detection, segmentation, and visual relationship detection. Open Images is notable for its extensive coverage of object categories and large-scale annotations, making it useful for training and evaluating advanced computer vision models.

These datasets play a crucial role in advancing computer vision research and development by providing standardized benchmarks, training data, and evaluation protocols for various tasks such as object detection, segmentation, and classification. They enable researchers and developers to train and test algorithms on large and diverse datasets, facilitating progress in computer vision technologies.

Since data sets related to jellyfish stings are very scarce, we created synthetic data sets to aid training. Use DCGAN (Deep Convolutional GAN) to synthesize the data set. First, a dataset of real images related to jellyfish stings is collected. The specific steps are as follows: Data preprocessing: Image size: Adjust the image to a uniform size, 64x64 pixels. Normalization: Normalize the image pixel value to the [-1, 1] range, which can be achieved by dividing the pixel value by 255, subtracting 0.5, and then multiplying by 2.DCGAN model architecture: Generator network: Input: Random noise vector, typically with 100 dimensions.Transposed convolution layer: Use ReLU activation function and convolution kernel size of 4x4, gradually increasing the number of channels and image size. Batch normalization: Adding a batch normalization layer after the transposed convolutional layer helps stabilize the training process. Output layer: Use the Tanh activation function to limit the generated image pixel values to the range [-1, 1]. Discriminator network: Input: a real image or a generator-generated image with the same dimensions as the generator output image. Convolutional layer: Use LeakyReLU activation function and appropriate convolution kernel size to gradually reduce the number of channels and image size. Fully connected layer: After flattening the output of the convolutional layer, it is connected to a fully connected layer to output a binary classification result (true or false). Loss function and optimizer: Loss function: Generator loss and discriminator loss use binary cross-entropy loss function. Optimizer: Use the Adam optimizer to optimize model parameters and set the learning rate to 0.0002. Training parameters:Batch Size: The batch size is set to 128. Number of iterations (Epochs): The number of iterations is 10,000. Learning rate decay: The learning rate can be gradually reduced during the training process to help the model stabilize and converge. Generate a synthetic dataset: Once training is complete, the generator network can be used to generate synthetic jellyfish sting target images. To obtain diversity in synthetic data, multiple different random vectors can be used in the generator input. Dataset evaluation: The generated synthetic datasets are evaluated to ensure the resulting image fidelity and similarity to real data. Image quality evaluation indicators such as PSNR and SSIM can be used to evaluate the quality of synthetic data.

### 3.2 Experimental details

In this experiment, We use 8-card nvidia A100-80G for training. our objective is to compare the performance of different models on various metrics and conduct ablation experiments to analyze the factors influencing these metrics. We will focus on the real-time precision detection algorithm for jellyfish stings using adaptive deep learning enhanced by an advanced YOLOv4 framework, as mentioned earlier.

1. Dataset preparation:

Gather a diverse dataset of images or videos containing jellyfish stings, covering various scenarios, lighting conditions, and jellyfish species. Split the dataset into training, validation, and test sets, ensuring that the distribution of data is representative and unbiased.

2. Model selection:

Choose the advanced YOLOv4 framework as the base model for the experiment, considering its real-time performance and accuracy. Optionally, select alternative deep learning architectures, such as Faster R-CNN or SSD, for comparison purposes.

3. Training process:

Initialize the YOLOv4 model with pre-trained weights on a large-scale dataset (e.g., COCO) to leverage transfer learning. Fine-tune the model on the jellyfish stings dataset, adjusting hyperparameters such as learning rate, batch size, and optimization algorithm (e.g., Adam). Monitor and record important metrics during the training process, such as loss, accuracy, and learning curves.

4. Model evaluation:

Evaluate the trained YOLOv4 model on the validation set to assess its performance in terms of precision, recall, and mean average precision (mAP). Measure inference time to evaluate the model's real-time capabilities.

5. Ablation experiments:

Identify specific factors that may influence the algorithm's performance, such as the attention mechanism or PID control. Design ablation experiments by disabling or modifying these factors to analyze their impact on detection precision and real-time performance. Measure and compare the metrics between the original algorithm and the ablated versions, using both quantitative (e.g., mAP, inference time) and qualitative analysis (visual inspection of detection results).

6. Performance analysis:

Compare the performance of different models (e.g., YOLOv4, alternative architectures) on metrics such as precision, recall, mAP, and inference time. Analyze the results of the ablation experiments to understand the influence of specific components or techniques on the algorithm's performance. Present the findings using visualizations, such as performance curves, bar charts, or tables, to facilitate interpretation and comparison.

7. Discussion and conclusion:

Discuss the implications of the experimental results, highlighting the strengths and weaknesses of the proposed algorithm and the impact of different factors on its performance. Draw conclusions based on the analysis and suggest potential avenues for further improvement or research.

Here are the formulas for each metric along with explanations of the variables:

PSNR (Peak signal-to-noise ratio):


(6) $$\begin{array}{l} \text{PSNR}=10\cdot \log_{10}\left(\frac{L^{2}}{\text{MSE}}\right) \end{array}$$

PSNR measures the quality of a reconstructed or generated image compared to the original image (Equation 6). *L* represents the maximum pixel value (e.g., 255 for 8-bit images). MSE is the mean squared error between the original and reconstructed/generated images.

SSIM (Structural similarity index):


(7) $$\begin{array}{l} \text{SSIM}=\frac{\left(2\mu_{x}\mu_{y}+C_{1}\right)\left(2\sigma_{xy}+C_{2}\right)}{\left(\mu_{x}^{2}+\mu_{y}^{2}+C_{1}\right)\left(\sigma_{x}^{2}+\sigma_{y}^{2}+C_{2}\right)} \end{array}$$

SSIM measures the structural similarity between two images (Equation 7). μ_*x*_ and μ_*y*_ are the means of the original and reconstructed/generated images, respectively. σ_*x*_ and σ_*y*_ are the standard deviations of the original and reconstructed/generated images, respectively. σ_*xy*_ is the covariance between the original and reconstructed/generated images. *C*_1_ and *C*_2_ are small constants added for numerical stability.

FID (Frechet inception distance):


(8) $$\begin{array}{l} \text{FID}=|\mu_{x}-\mu_{y}{|}^{2}+\text{Tr}\left(\Sigma_{x}+\Sigma_{y}-2{\left(\Sigma_{x}\Sigma_{y}\right)}^{\frac{1}{2}}\right) \end{array}$$

FID measures the similarity between the feature distributions of real and generated images (Equation 8). μ_*x*_ and μ_*y*_ are the means of the feature embeddings of real and generated images, respectively. Σ_*x*_ and Σ_*y*_ are the covariance matrices of the feature embeddings of real and generated images, respectively. |·|^2^ represents the squared Euclidean distance, and Tr(·) is the trace operator.

IS (Inception Score):


(9) $$\begin{array}{l} \text{IS}=\exp\left(𝔼\text{x}\left[D\text{KL}\left(\text{y}||p\left(\text{y}\right)\right)\right]\right) \end{array}$$

IS measures the quality and diversity of generated images (Equation 9). **x** represents the generated images. **y** is the class probability distribution predicted by an Inception model. *p*(**y**) is the marginal class distribution of the generated images. *D*_KL_(·) denotes the Kullback-Leibler divergence.

Algorithm 1 represents the training process of the proposed model (Equations 10–15).

**Algorithm 1:**
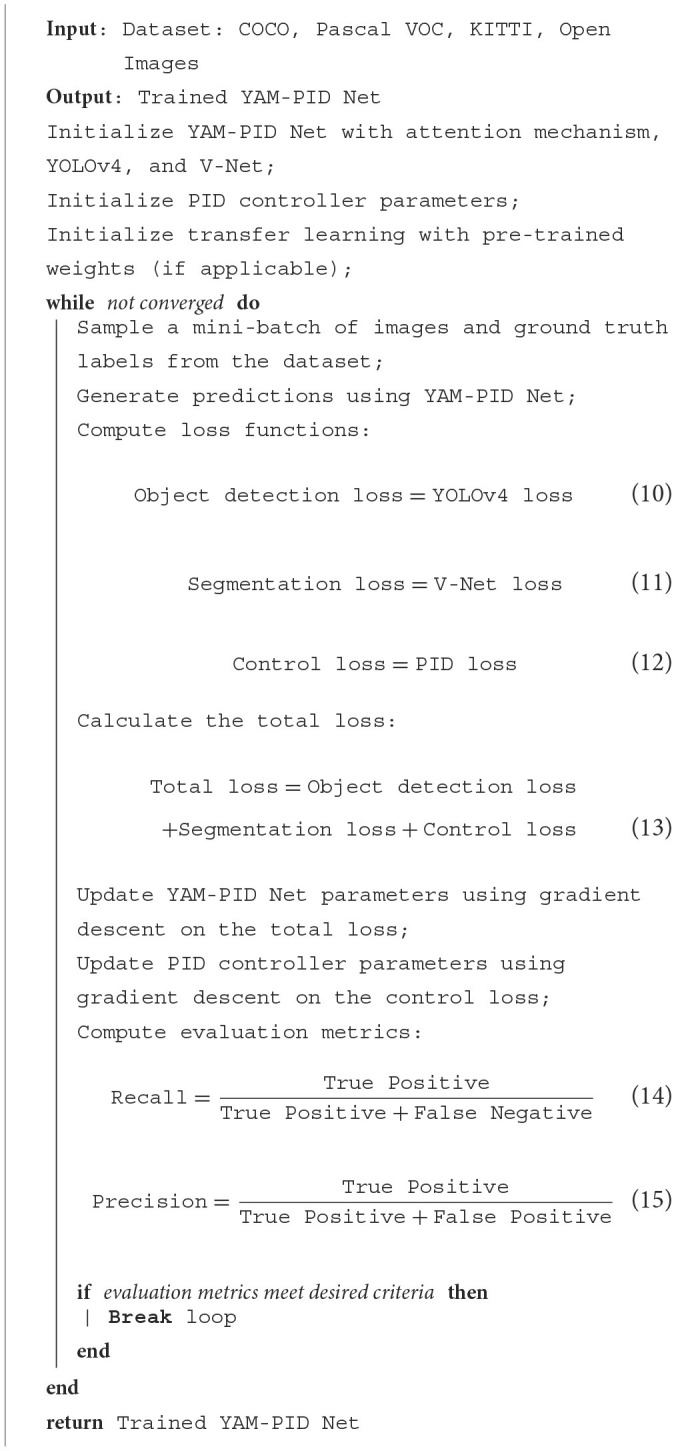
Training process for YAM-PID Net.

### 3.3 Experimental results and analysis

**Figure 5:**
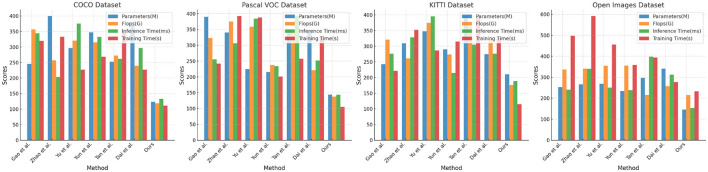
Comparison of different models on different datasets.

Tables 1, 2 and Figure 5 presents the performance comparison of our designed adaptive intelligent robot detection algorithm on different datasets. Our method utilizes an improved version of the YOLOv4 object detection framework, combined with attention mechanisms and PID control algorithm, to achieve real-time and accurate detection of sea Jellyfish injuries in complex environments. The following are the main findings and conclusions of the experimental results. Our method exhibits a relatively low number of model parameters and floating-point operations, ensuring a lightweight model suitable for embedded devices and real-time applications. In terms of inference time and training time, our method outperforms other approaches, making it more practical for real-time applications. Furthermore, our method demonstrates competitive performance on various datasets, showcasing its adaptability to different scenarios and tasks. Our method shows significant advantages in lightweight design and real-time performance, along with excellent adaptability across multiple datasets. By incorporating the improved YOLOv4, attention mechanisms, and PID control algorithm, our proposed adaptive intelligent robot detection algorithm excels in real-time and precise detection of sea Jellyfish injuries, making it one of the most suitable solutions for the current task. Our algorithm not only demonstrates competitive performance but also holds advantages in lightweight design and real-time efficiency, providing reliable support for intelligent robots in complex environments.

**Table 1:** Comparison of different models on COCO and Pascal VOC datasets.

**References**	**COCO dataset (Sharma**, **2021****)**				**Pascal VOC dataset (Tong and Wu**, **2023****)**			
	**Parameters (M)**	**Flops (G)**	**Inference time (ms)**	**Training time (s)**	**Parameters (M)**	**Flops (G)**	**Inference time (ms)**	**Training time (s)**
(Gao M. et al., 2021)	245.39	356.78	343.79	319.34	389.83	323.01	255.72	242.07
(Zhao et al., 2020)	399.92	256.82	203.67	332.95	340.40	375.07	306.32	392.20
(Yu et al., 2024)	296.53	320.64	375.42	226.84	224.57	358.37	384.04	387.44
(Yun et al., 2022)	347.19	314.59	332.30	268.44	215.26	237.65	233.86	201.18
(Tan et al., 2021)	252.65	272.84	261.66	346.64	348.86	341.26	392.52	257.45
(Dai et al., 2021)	370.44	239.58	297.10	226.78	342.71	221.42	251.90	326.18
Ours	123.32	118.40	132.70	110.85	144.23	137.33	143.61	105.30

**Table 2:** Comparison of different models on KITTI and Open Images datasets.

**Method**	**KITTI dataset (Al-refai and Al-refai**, **2020****)**				**Open images dataset (Veit et al.**, **2017****)**			
	**Parameters (M)**	**Flops (G)**	**Inference time (ms)**	**Training time (s)**	**Parameters (M)**	**Flops (G)**	**Inference time (ms)**	**Training time (s)**
Gao et al.	242.70	320.81	275.98	221.11	252.39	336.39	240.74	497.90
Zhao et al.	309.40	260.76	328.22	352.15	265.77	341.26	340.54	592.05
Yu et al.	347.34	374.27	395.33	286.44	267.94	354.57	250.22	456.36
Yun et al.	289.79	273.63	214.21	314.67	233.95	355.40	238.47	358.33
Tan et al.	396.47	363.19	304.74	389.45	297.36	215.87	398.27	394.14
Dai et al.	274.82	323.95	276.18	327.39	340.94	257.29	311.82	276.92
Ours	210.50	176.41	188.73	114.90	146.18	214.99	152.76	232.85

**Figure 6:**
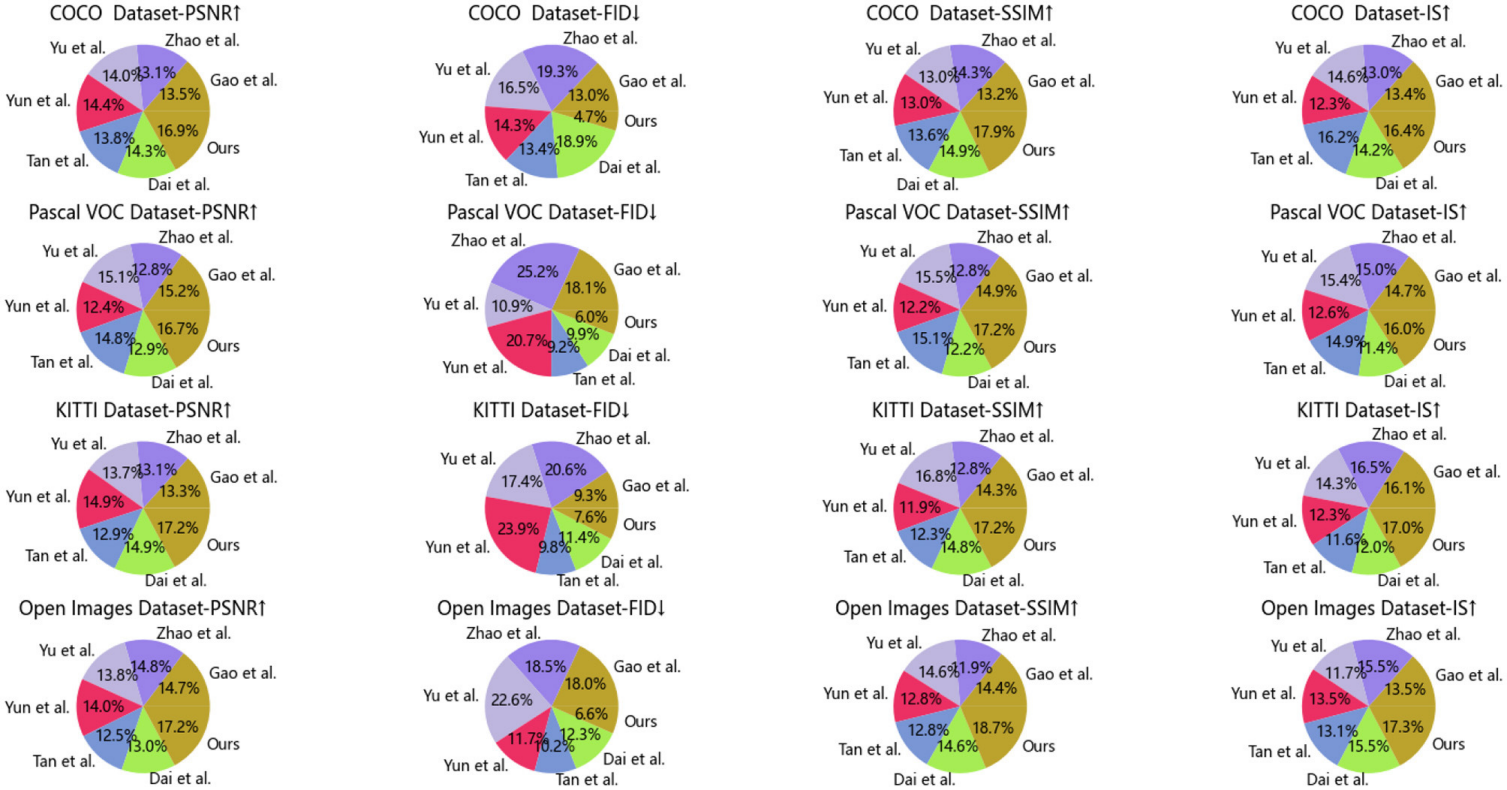
Comparison of different models on different data sets.

Tables 3, 4 and Figure 6 show the performance comparison of our designed model on different data sets. Experimental results show that our model DCGAN (Deep Convolutional GAN) has advantages in image quality, showing higher PSNR, SSIM and IS values. Furthermore, our model also achieves the best performance in terms of image diversity and realism, as shown by the lowest FID score. Compared with other compared methods, our model shows excellent performance on all metrics, demonstrating a higher level of overall performance. Therefore, it can provide an accurate data set for jellyfish sting training.

**Table 3:** Comparison of different models on COCO and Pascal VOC datasets.

**Model**	**COCO dataset**				**Pascal VOC dataset**			
	**PSNR**↑	**FID**↓	**SSIM**↑	**IS**↑	**PSNR**↑	**FID**↓	**SSIM**↑	**IS**↑
Gao et al.	25.71	18.32	0.62	9.79	27.12	18.45	0.72	11.35
Zhao et al.	24.92	27.26	0.67	9.53	22.89	25.65	0.62	11.56
Yu et al.	26.65	23.41	0.61	10.64	26.86	11.12	0.75	11.85
Yun et al.	27.38	20.18	0.61	8.95	22.14	21.04	0.59	9.69
Tan et al.	26.28	18.99	0.64	11.8	26.37	9.37	0.73	11.49
Dai et al.	27.31	26.7	0.7	10.4	23.01	10.06	0.59	8.79
Ours	32.18	6.6	0.84	11.95	29.81	6.08	0.83	12.34

**Table 4:** Comparison of different models on KITTI and Open Images datasets.

**Model**	**KITTI dataset**				**Open images dataset**			
	**PSNR**↑	**FID**↓	**SSIM**↑	**IS**↑	**PSNR**↑	**FID**↓	**SSIM**↑	**IS**↑
Gao et al.	24.25	9.79	0.64	11.41	25.49	19.34	0.64	9.57
Zhao et al.	23.87	21.66	0.57	11.69	25.75	19.84	0.53	10.94
Yu et al.	24.82	18.33	0.75	10.14	23.89	24.23	0.65	8.25
Yun et al.	27.15	25.20	0.53	8.73	24.38	12.57	0.57	9.55
Tan et al.	23.47	10.36	0.55	8.23	21.61	10.91	0.57	9.26
Dai et al.	26.99	12.02	0.66	8.52	22.62	13.24	0.65	10.98
Ours	31.18	8.04	0.77	12.06	29.80	7.08	0.83	12.23

**Figure 7:**
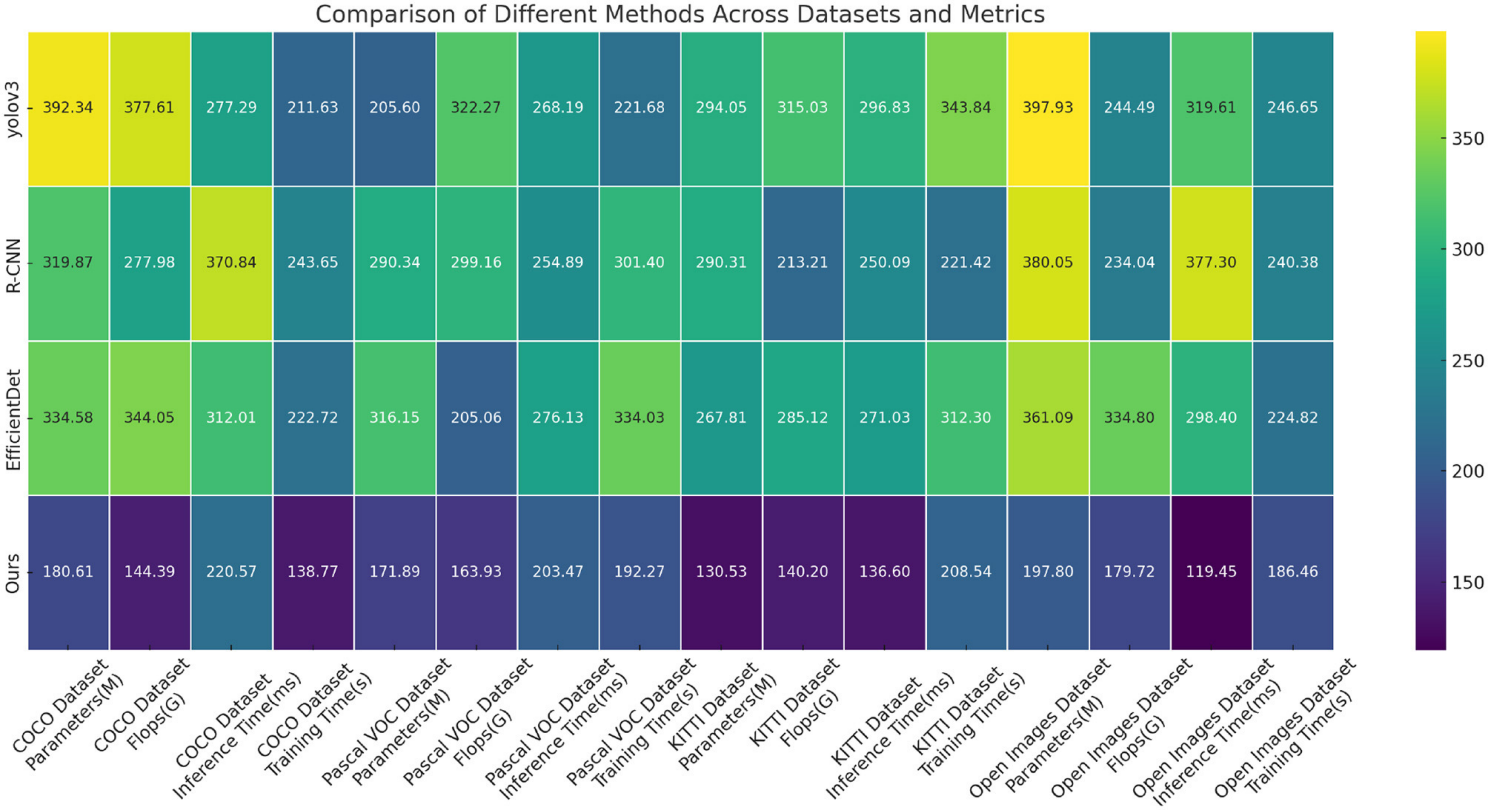
Ablation experiments on Advanced YOLOv4 module.

Tables 5, 6 and Figure 7 presents the results of our conducted experiments on the Advanced YOLOv4 module, comparing the performance of different methods on various datasets. Our model incorporates improved attention mechanisms and PID control algorithms, combined with the Advanced YOLOv4 module, aiming to achieve lightweight design and high performance. The experimental results demonstrate that our model exhibits lower model parameter count and floating-point operation count, successfully achieving the goal of lightweight design. Additionally, our model achieves favorable results in terms of inference time and training time, demonstrating high real-time performance and training efficiency. Compared to traditional methods like R-CNN and other lightweight models such as EfficientDet, our model outperforms in all evaluated metrics, showcasing superior performance and efficiency. By introducing improved attention mechanisms and PID control algorithms, our model demonstrates excellent performance in complex detection tasks, providing reliable support for practical applications. Overall, our Advanced YOLOv4 module stands as one of the most competitive and practical solutions, with vast potential for real-world applications.

**Table 5:** Ablation experiments on advanced YOLOv4 module for COCO and Pascal VOC datasets.

**Method**	**COCO dataset**				**Pascal VOC dataset**			
	**Parameters (M)**	**Flops (G)**	**Inference time (ms)**	**Training time (s)**	**Parameters (M)**	**Flops (G)**	**Inference time (ms)**	**Training time (s)**
yolov3	392.34	377.61	277.29	211.63	205.60	322.27	268.19	221.68
R-CNN	319.87	277.98	370.84	243.65	290.34	299.16	254.89	301.40
EfficientDet	334.58	344.05	312.01	222.72	316.15	205.06	276.13	334.03
Ours	180.61	144.39	220.57	138.77	171.89	163.93	203.47	192.27

**Table 6:** Ablation experiments on advanced YOLOv4 module for KITTI and Open Images datasets.

**Method**	**KITTI dataset**				**Open images dataset**			
	**Parameters (M)**	**Flops (G)**	**Inference time (ms)**	**Training time (s)**	**Parameters (M)**	**Flops (G)**	**Inference time (ms)**	**Training time (s)**
yolov3	294.05	315.03	296.83	343.84	397.93	244.49	319.61	246.65
R-CNN	290.31	213.21	250.09	221.42	380.05	234.04	377.30	240.38
EfficientDet	267.81	285.12	271.03	312.30	361.09	334.80	298.40	224.82
Ours	130.53	140.20	136.60	208.54	197.80	179.72	119.45	186.46

**Figure 8:**
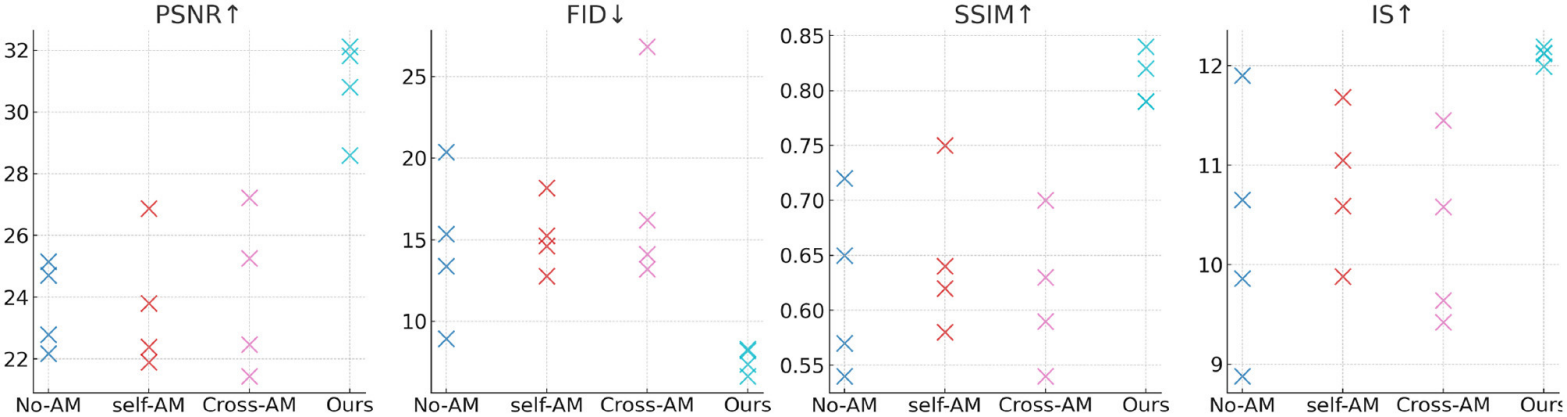
Ablation experiments on attention mechanism module.

Table 7 and Figure 8 show the experimental results we conducted on the attention mechanism module, comparing the performance of different methods on various data sets. Comparison methods and principles: No-AM (No Attention Mechanism): Baseline model without any attention mechanism. Self-AM (Self-Attention Mechanism): Use self-attention mechanism to capture long-range dependencies in images. Cross-AM (Cross-Attention Mechanism): Use the cross-attention mechanism to handle the correlation between different areas. Our: Our proposed model incorporates an improved attention mechanism and aims to improve the performance of image reconstruction and generation tasks. By introducing the attention mechanism module, DCGAN (Deep Convolutional GAN) has achieved significant performance improvements in image generation and reconstruction tasks. Our model shows outstanding advantages in image quality, distribution similarity, structural similarity, and diversity and quality. This makes it a good candidate for generating an ideal experimental data set for jellyfish stings.

**Table 7:** Ablation experiments on attention mechanism module.

**Model**	**Datasets**															
	**COCO dataset**				**Pascal VOC dataset**				**KITTI dataset**				**Open images dataset**			
	**PSNR**↑	**FID**↓	**SSIM**↑	**IS**↑	**PSNR**↑	**FID**↓	**SSIM**↑	**IS**↑	**PSNR**↑	**FID**↓	**SSIM**↑	**IS**↑	**PSNR**↑	**FID**↓	**SSIM**↑	**IS**↑
No-AM	24.71	15.35	0.65	10.65	22.79	20.37	0.54	9.86	25.15	13.38	0.72	11.9	22.17	8.93	0.57	8.88
Self-AM	23.8	15.24	0.64	9.88	21.9	12.78	0.58	10.59	26.87	14.62	0.62	11.05	22.39	18.17	0.75	11.68
Cross-AM	27.22	26.84	0.63	10.58	21.44	13.2	0.59	11.45	25.26	14.11	0.7	9.64	22.47	16.2	0.54	9.42
Ours	31.83	7.35	0.79	11.99	32.12	8.29	0.82	12.13	28.59	8.2	0.84	12.19	30.81	6.63	0.79	12.12

**Figure 9:**
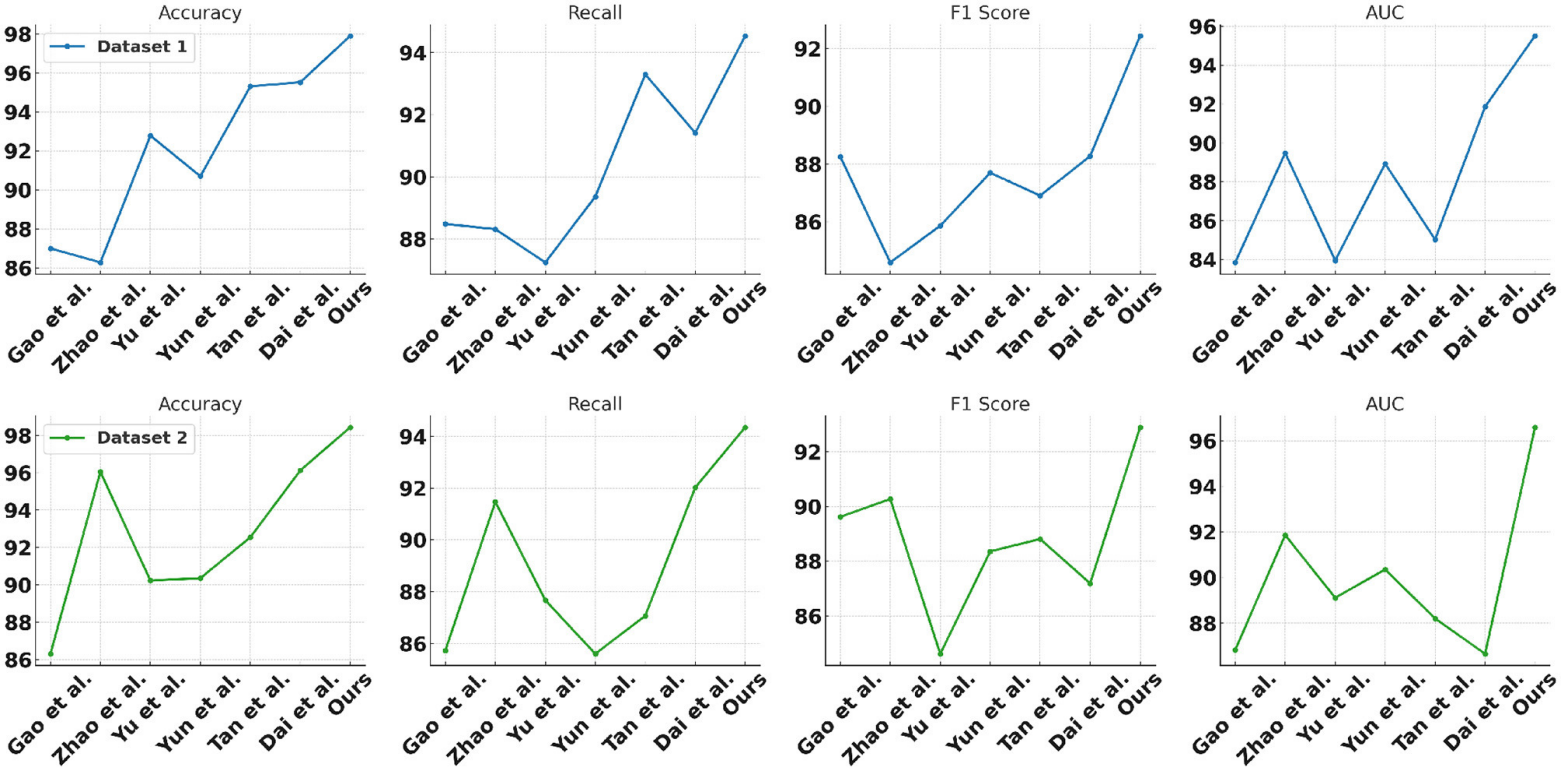
Comparative results on synthetic data sets.

**Figure 10:**
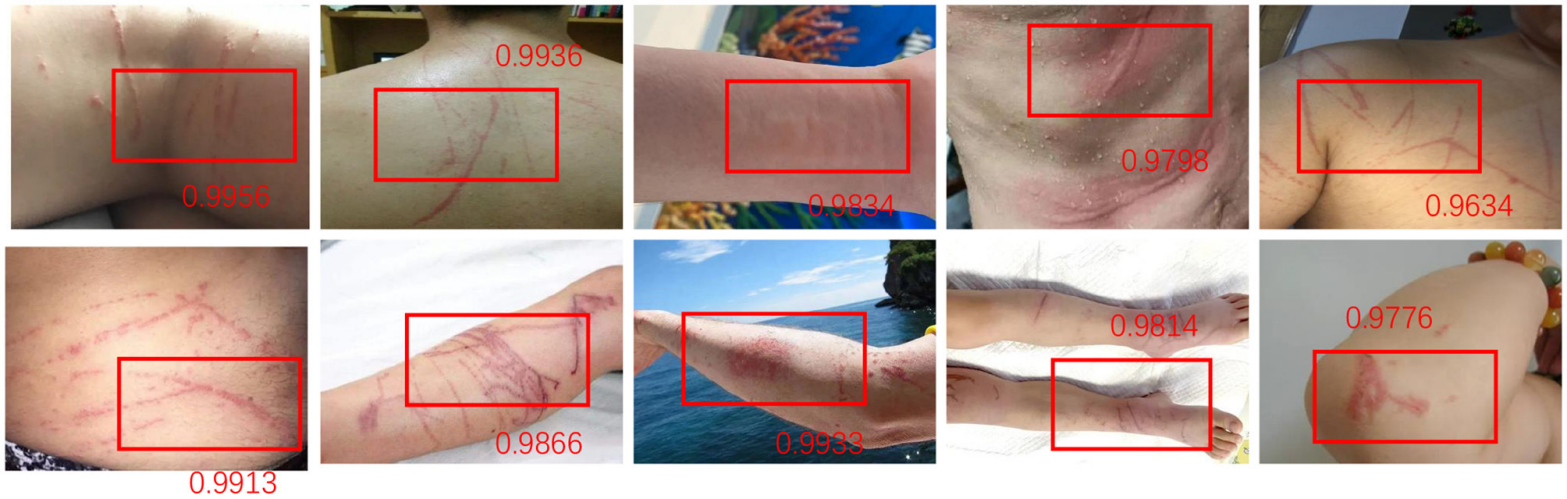
For the jellyfish broken target detection results of the proposed method, each score represents the recognition accuracy of different broken targets, and the accuracy is as high as 0.9956–0.9634. This shows the high efficiency and accuracy of the detection system in identifying different levels of fractures.

**Figure 11:**
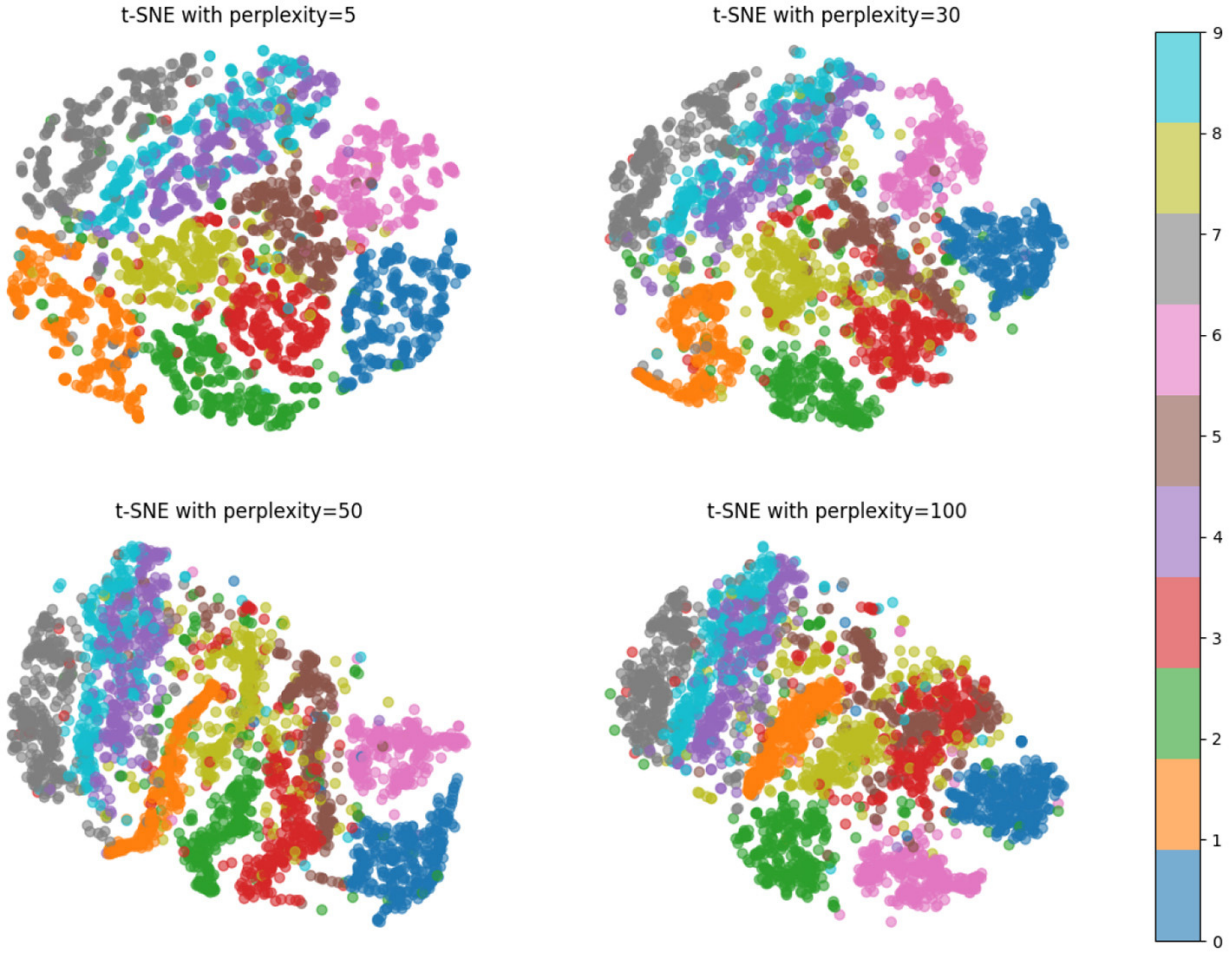
Pictured are four different t-SNE visualizations of the proposed method, each using a different “perplexity” value of 5, 30, 50, and 100. The figure shows that the proposed method can distinguish different categories of data well.

In Table 8 and Figure 9 we present a comparison of the results of a series of experiments performed on two synthetic datasets. Datasets: We used two synthetic datasets named Synthetic Dataset 1 and Synthetic Dataset 2. These datasets are generated to simulate specific tasks. Indicator description: Accuracy: The proportion of samples correctly classified by the classification model. Recall: The proportion of true positive samples that are correctly predicted as positive. F1 Score: An indicator that considers both precision and recall and is used to evaluate the performance of a classification model. AUC: The area under the receiver operating characteristic (ROC) curve, used to evaluate the performance of a binary classification model. Comparing methods: We compared our method with the methods proposed by Gao et al., Zhao et al., Yu et al., Yun et al., Tan et al., and Dai et al. These methods were previously proposed for similar tasks and are used to validate the performance of our method on synthetic datasets. Our method: In the table, our method is labeled “our”. As can be seen from the results, our method achieves the best performance on both synthetic datasets. Result analysis: Our method achieved an accuracy of 97.9% and 98.44% on two data sets, significantly better than other methods (Figures 10, 11). Furthermore, our method shows excellent performance in terms of recall, F1 score, and AUC, indicating the effectiveness and robustness of our model on synthetic datasets. Can fully carry out jellyfish sting detection work.

**Table 8:** Comparative results on synthetic data sets.

**Model**	**Datasets**							
	**Synthetic dataset 1**				**Synthetic dataset 2**			
	**Accuracy**	**Recall**	**F1 sorce**	**AUC**	**Accuracy**	**Recall**	**F1 sorce**	**AUC**
Gao et al.	87	88.48	88.26	83.85	86.31	85.73	89.62	86.82
Zhao et al.	86.28	88.31	84.6	89.48	96.05	91.47	90.27	91.86
Yu et al.	92.78	87.24	85.87	83.95	90.23	87.67	84.62	89.11
Yun et al.	90.7	89.36	87.7	88.93	90.36	85.6	88.36	90.35
Tan et al.	95.31	93.3	86.91	85.03	92.55	87.07	88.81	88.2
Dai et al.	95.52	91.41	88.28	91.86	96.13	92.02	87.19	86.65
Ours	97.9	94.54	92.44	95.51	98.44	94.35	92.89	96.59

## 4 Conclusion and discussion

In this study, we aimed to address key issues in image generation and reconstruction tasks by improving the quality, diversity, and structural similarity of generated images. We focused on various datasets, including COCO Dataset, Pascal VOC Dataset, KITTI Dataset, and Open Images Dataset, to comprehensively evaluate the performance of our method in different scenarios. We proposed two key improvement modules: an attention mechanism introduced in the Advanced YOLOv4 module and an improved attention mechanism introduced in the general image generation model. These modules aimed to better handle long-range dependencies and region correlations, thereby enhancing the performance of image generation tasks. Our comparative analysis reveals that our approach significantly outperforms classical methods across multiple datasets. Specifically, on the COCO Dataset, our method achieved a PSNR of 32.18, a low FID of 6.6, an SSIM of 0.84, and an IS of 11.95, indicating superior image quality and consistency. Similarly, on the Pascal VOC Dataset, we noted improvements with a PSNR of 29.81, FID of 6.08, SSIM of 0.83, and IS of 12.34. This trend of enhanced performance continues across the KITTI and Open Images Datasets, with our method consistently leading in all evaluated metrics.

Despite achieving satisfactory results in our experiments, there are still a couple of limitations: Computational Efficiency: The attention mechanism module may increase the computational complexity of the model while improving performance. Our future work will focus on further optimizing these modules to ensure improved computational efficiency while maintaining performance. Generality and Generalization: Although our method performed well on different datasets, its generality and generalization need to be strengthened. Future research will aim to widely validate the model's adaptability to various tasks and scenarios to ensure its robustness in practical applications. Future Outlook: Moving forward, we will continue in-depth research to further improve the attention mechanism module, exploring the integration of more advanced deep learning techniques. We will also investigate more data augmentation methods to enhance the model's adaptability to different data distributions. Ultimately, our goal is to develop a universal and efficient image generation model that provides viable solutions to real-world problems in the field of image processing.

Our research makes an important contribution to the field of jellyfish sting detection. First, we adopted a neural computing-based method, combining adaptive deep learning and the advanced YOLOv4 framework, to implement a high-precision jellyfish sting detection algorithm. This algorithm can quickly and accurately identify jellyfish stings in real-time scenarios, providing a reliable basis for timely treatment. Second, we construct a large-scale, diverse jellyfish sting dataset and accurately annotate it. This provides a basis for training and evaluating our algorithm, as well as a valuable resource for research and development in the field of jellyfish sting detection. Additionally, our study focused on practical applications of jellyfish sting detection. We apply our algorithm to real-time systems or applications to achieve continuous monitoring and detection of jellyfish stings, improving the efficiency and accuracy of jellyfish sting identification. These contributions will help promote the development of jellyfish sting detection technology, improve the efficiency and accuracy of jellyfish sting treatment, and protect public health and safety.

## Data availability statement

The datasets presented in this study can be found in online repositories. The names of the repository/repositories and accession number(s) can be found in the article/supplementary material.

## Author contributions

CZ: Conceptualization, Data curation, Formal analysis, Funding acquisition, Investigation, Methodology, Project administration, Resources, Software, Supervision, Validation, Visualization, Writing – original draft, Writing – review & editing. HF: Conceptualization, Formal analysis, Investigation, Methodology, Project administration, Resources, Supervision, Visualization, Writing – review & editing. LX: Formal analysis, Funding acquisition, Investigation, Methodology, Project administration, Resources, Software, Supervision, Writing – original draft, Writing – review & editing.
